# Remarks on Dr. Reynolds' Case of Restoration after Necrosis, and Removal of Inferior Maxilla

**Published:** 1878-08

**Authors:** J B. Hodgkin


### ARTICLE VII.

### *Remarks on the Cure reported by Dr. Geo. B. Reynolds, (Virginia Medical Monthly, June, 1878,)—the Restoration after Necrosis, and Removal of the lower Maxilla.*

BY PROF. HODGKIN.

[The article in question was published in a former No. of this Journal.—Eds. Am. Jour. of Dental Science.]

I now turn the case over to my friend and your teacher, Prof. Hodgkin, who will tell you what can be done toward replacing with artificial substitutes, the boy's lost teeth.

Prof. Hodgkin:—I am sure that I can add little of interest to this case by speaking now, after the talk which Dr. Reynolds has given you; and indeed I am not sufficiently familiar with its details to do so. He has said all that can be said about it, and it only remains for me to join with you in admiration, of this conservative Surgery of his and of his associate, Dr. Trist, and to tell you how to make a lower artificial set of teeth for this boy.

But I cannot help calling your attention to the wonderful reproductive power of nature, as evidenced in this case. It shows you as no other evidence probably could, the function of the periosteum, which in this case was left intact, and to remark upon the nice discrimination which kindly nature shows, when her best efforts are not thwarted by man's bungling. How wonderful is the conservatism which allows a dead bone to slough away from its periosteal attachments, to sever its connection with the ligaments, which you know are so firmly secured as to permit absolute fracture of the bone before this will tear away; to preserve, although bathed in pus for months, this delicate membrane around the bone, and leave its functional activity unimpaired. I cannot express how I admire all this, and wonder at it. If any anchylosis are present, if any notable deformity, the case would not be so striking; but here we can almost perfect reproduction, for with the exception that the bone seems to be a little smaller, and you see that the chin is a trifle short, and the teeth as a matter of course inspiring, all seems perfect.

Of course we understand that in this case, the care of the teeth is final, at least so far as the teeth which were erupted or in process of eruption, are concerned This boy has a single lower molar—apparently the second—which has erupted since this operation on the right side. The wisdom tooth may and probably will melt in due course of time. But the germ of the wisdom tooth, on the left side, must have been removed with the bone, as the whole of the jaw, including the ramous, was dissected out.

There is one other point, I wish to bring out. You observe that this jaw, in all respects resembles one rendered edentulous by accident,—it is just like one from which you have removed all the teeth and in which the ordinary absorbtion and disappearance of the alveolus has occurred; and yet this jaw has not been subject to such accident, but is simply a maxilla, minus teeth. It teaches us that the alveoli are merely incidents of dentition, and are in no way inseparably associated with the development of the bone. They have no more to do with the progress of the growth of the maxillæ than the crusts of earth, which are pushed up around the plant erupting from the earth has to do with the shape of the earth. It proves that the teeth are in no way concerned in the development of the jaw—do not aid in its expansion,—for here we have a jaw of normal width, developed without teeth. The shortening of the chin, which is slight, can be accounted for by the contraction of the muscles, and the only wonder is that it is not greater. Here in this show-case are casts of the upper and lower maxillæ, of a man who assures us that he had neither temporary or permanent teeth between his lower canines, and yet you see that the space between these canines is amply wide for the accommodation of such teeth were they present;—his jaw was as fully developed at this point without teeth as with them.

This subject may thus throw some light on the vexed question of the reproduction, or the failure of reproduction, of the lost alveolus,—lost by wasting, or other cause. It may teach us that, as this second bone was without alveoli, that nature does not attempt the restoration of this process when lost, and certainly we see in practice that this is true, at least so far as the thinner and now delicate portions of it are concerned. I do not believe that the alveolus of a tooth is ever restored; and I am not even sure that the septum of bone between the molars, when removed by accident or disease, grows again. I am watching a case now with great interest, in which the septum of bone between the first and second superior molars has wasted in consequence of, as I think, an incautious use of arsenic, (I did not find it there!)—watching to see if it grows again. I am anxious that it should, but hardly believe it.

This boy must have a lower piece made for him. I rather incline to the opinion, that as this bone may grow still more, it would be well to wait awhile until the fullest possible development takes place. We have then, what is rare in replacement, the work of making a lower set to articulate with the upper natural teeth. I recollect to have seen but one other case, where this was necessary, as usually the case is reversed.

